# Comparison of Three Prototypes of PPG Sensors for Continual Real-Time Measurement in Weak Magnetic Field †

**DOI:** 10.3390/s22103769

**Published:** 2022-05-16

**Authors:** Jiří Přibil, Anna Přibilová, Ivan Frollo

**Affiliations:** Institute of Measurement Science, Slovak Academy of Sciences, 841 04 Bratislava, Slovakia; umerapri@savba.sk (A.P.); umerollo@savba.sk (I.F.)

**Keywords:** photoplethysmography optical sensor, wearable sensor, PPG signal processing

## Abstract

This paper is focused on investigation of three developed prototypes of sensors based on the photoplethysmography (PPG) principle for continual measurement of the PPG signal in the magnetic field environment with the inherent radiofrequency and electromagnetic disturbance. The tested prototypes differ in the used optical part of the PPG sensor and their working mode, control unit, power supply, and applied Bluetooth (BT) communication methods. The main aim of the current work was motivated by finding suitable and universal parameter settings for PPG signal real-time recording in different working mode conditions. Comparative measurements in laboratory conditions by certified commercial pulse oximeter and blood pressure monitor (BPM) devices show good stability and proper accuracy of finally determined heart rate values. The supplementary investigation certifies the necessity of the placement of the pressure cuff of the BPM device on the opposite arm than the tested PPG sensor. Measurement experiments inside the scanning area of the running weak field magnetic resonance scanner verify proper function and practical usability of sensed PPG signals for further processing and analysis in all three prototype cases. Additional testing shows that the BT transmission in the scanning area has no visible influence on the quality of the finally obtained scanner images.

## 1. Introduction

During a non-invasive medical examination in a magnetic resonance imaging (MRI) scanner, patients are exposed to the mechanical vibration of the gradient-coil system and the resulting acoustic noise. These factors have a negative influence on the subject’s cardiovascular system and evocate a mental stress [1] that can be detected and evaluated by continual monitoring of blood pressure (BP) and heart rate (HR) parameters. This monitoring is often carried out using wearable sensors based on the photoplethysmography (PPG) [2,3]. The PPG signals can be sensed and recorded from various body positions: finger, ear, wrist, arm, neck, nose, chest, and forehead [4], so different practical realizations of PPG sensors are necessary. Various parameters identifying the physiological and mental stress can be derived from the PPG signal: pulse transit time [2], pulse wave velocity [5], blood oxygen saturation, cardiac output [6], etc. MRI tomography is also used to obtain vocal tract shapes during the articulation of speech sounds for the articulatory synthesis [7]. Here, the stress-evoked vocal cord tension has an influence on the recorded speech signal [8] by modifying its supra-segmental and spectral features; therefore, it can bring about errors and inaccuracy in calculation of 3D models of the human vocal tract [9]. Analysis, mapping, detection, and evaluation of this negative effect were the basis of our previous [10,11] as well as present [12] research investigation. For this reason, three prototypes of PPG sensors with real-time Bluetooth (BT) transmission were subsequently developed. Due to the static magnetic field inside the MRI scanner accompanied by an inherent radiofrequency (RF) and electromagnetic (EM) disturbance [13], a special realization of sensors must be used for PPG signal pickup.

Our present work was practically motivated by seeking suitable and universal parameter settings for PPG signal real-time recording in different working mode conditions including proper function in a weak magnetic field environment. Before starting this development, we had already tested commercial fitness bracelets and smart watches. These types of devices work in the reflectance mode and product HR parameter values that are not very precise and are not designed for function in these conditions (in addition with inherent RF and EM disturbance). Next, majority of these commercial devices are not able to send the PPG signal samples to an external device (the HR values only). For the purpose of this study, we need to evaluate fast and slow changes in the heart rate as well as changes in the width of systolic/diastolic pulses to determine special PPG signal properties for stress or pain detection. A possible solution seems to be using an oximeter device working in the transmission mode—more expensive types enable real-time visualization of the sensed PPG wave. These devices have better resistance to interference, but their practical problem originates from the experimental arrangement which must be fulfilled: a PPG/oximeter device placed inside the scanning area of the MRI tomograph must communicate wirelessly with an external device which is located outside the shielding cage of the whole MRI equipment. All oximeters, which we had at our disposal for preliminary testing, had a poor, unstable BT data connection thru the metal cage, often the connection cannot be established whatsoever. Therefore, we finally decided to develop new sensor prototypes which can solve all problems and limitation conditions. Prior to full experimental usage of the developed prototypes of PPG sensors, the evaluation of precision and functional stability by comparative measurements must be performed in the laboratory as well as in real conditions inside the running MRI device. In our last work presented in [12], two prototypes of wearable PPG sensors working in a reflectance mode were compared. The comparative measurements performed with the certified commercial pulse oximeter (POXI) device in the normal laboratory conditions confirm sufficient stability and precision of the determined HR values. Additional analysis of the influence of the used smoothing method on the finally obtained PPG signal properties was also realized in the previous work. First-step measurements inside the scanning area of the working MRI device with low magnetic field verify practical functionality of both PPG sensors and their potential usability in further measurements.

The current extension (improvements) consists in the basic fact that this article compares three developed PPG sensor prototypes where the third one works in a transmittance mode and is practically unwearable. It means that all three investigated prototypes differ in the used optical part of the PPG sensor working in reflectance/transmittance modes, control units based on Arduino boards [14], power supplies, used BT modules, and communication standards—but all three prototypes enable functioning in the low magnetic field environment with RF and EM disturbance. The first part of the current work is focused on description and comparison consisting of following 4 points: (1) characterization of types of PPG waveforms and their basic features; (2) detailed description of a method practically used for real-time calculation of HR values and determination of PPG signal properties enabling their statistical analysis in the frame of post-processing operations; (3) summarization of common characteristics and differences of three tested PPG sensors; (4) basic description of created application for Windows platform which controls real-time sensing of the PPG wave, signal preprocessing (filtering), determination of HR values, and final storing in a Wave format. In the second part, this study comprises following 6 measurement experiments: (1) measurement of the sensor’s mean direct current using different power supply of 5/3.7 V in the 3 operation modes; (2) the calibration measurement of determined HR values from PPG signals sensed by all 3 tested PPG sensors together with parallel measurements by a certified blood pressure monitor (BPM) and OXI devices; (3) an analysis of necessity of proving a pressure effect of the inflated BPM cuff by its quantification and mapping during the simultaneous measurement of the PPG signal consecutively picked up from fingers of both hands; (4) testing of the stability and quality of BT connection between the tested PPG sensor located inside the scanning area of the MRI device and the control device located outside the shielding cage; (5) an analysis of the influence of the BT transmission in the scanning area on the quality of the finally obtained MR images; (6) a verification of practical functionality of all 3 tested PPG sensor prototypes in the scanning area of the running open-air MRI scanner based on a static field with magnetic induction up to 0.2 T.

## 2. Methods

### 2.1. Photoplethysmography—Types, Basic Properties, and Principles of Sensing

The photoplethysmography is a noninvasive optical method used to monitor pulsatile blood flow in arteries. A typical PPG waveform consists of two components: a non-pulsatile or direct current (DC) component originates from light absorption in the non-pulsating tissue. The superimposed pulsatile or alternating current (AC) component is generated due to light absorption in the pulsatile arterial blood and synchronously varies with the beating of the heart, so it is a simple tool for the heart rate (HR) monitoring. The magnitude of the AC component is much smaller—typically about 2% of the DC component. The PPG sensors can be placed at various body locations, for example, finger, ear, wrist, arm, chest, face. Apart from the HR and HR variability, other physiological parameters can be estimated from the PPG signal, for example, arterial blood oxygen saturation denoted as SpO_2_ when measured by the POXI device. Here two PPG signals are picked up and SpO_2_ is calculated using the ratio of normalized AC components of both signals [3].

**Figure 1:**
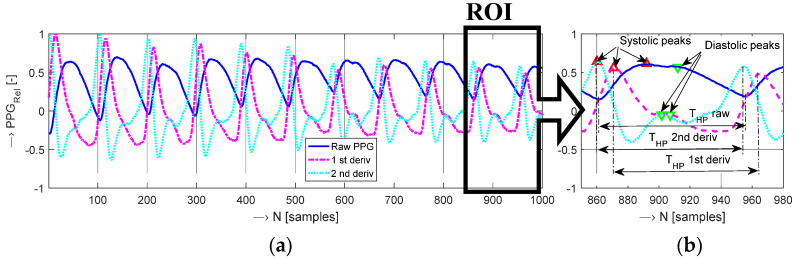
Example of three basic types of photoplethysmograms: (**a**) 1-k sample of the raw photoplethysmogram together with its first/second derivatives, (**b**) detailed 100 sample ROI around the 10th PPG cycle together with determined maxima corresponding to systolic and diastolic peaks and a denoted heart pulse period *T*_HP_.

A crucial step in the PPG pulse wave analysis is calculation of signal derivatives that may be used for determination of pulse wave features. The original pulse wave (raw photoplethysmogram—PTG) as well as its first derivative (called as velocity photoplethysmogram—VPG) and its second derivative (known as acceleration photoplethysmogram—APG) can be used to identify points of interest [15]. In all PPG signals, each photoplethysmogram cycle contains two local maxima representing systolic and diastolic peaks. These peaks provide valuable information about the pumping action of the heart—see an example in Figure 1 together with a detailed region of interest (ROI) with denoted heart pulse periods (*T*_HP_).

As documented in Figure 1, the raw PPG signal does not seem suitable for local maxima determination. On the other hand, the first- and the second-order derivatives of this PPG signal are more informative due to more pronounced local extremes. The PPG waveform for the purpose of classification or prediction processes could also be used in the form of an image in a similar way as this approach is applied in the case of real-time electrocardiogram processing [16].

**Figure 2:**
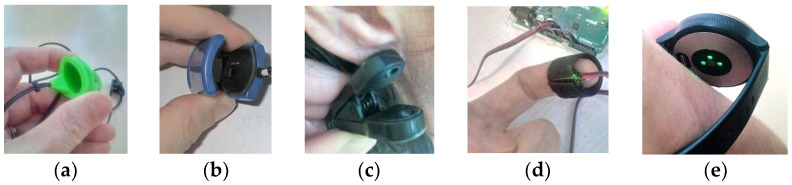
Examples of practical realizations of optical parts of a PPG sensor: (**a**) rubber finger ring [21], (**b**) plastic finger clip [20], (**c**) plastic ear clip [22], (**d**) reflectance PPG sensor with analogue interface fixed on an index finger by an elastic ribbon, (**e**) back side of the Garmin watch VivoActive 3 with three green LEDs and one PD inside—loosely put on a wrist [23].

A conventional PPG sensor consists of a light emitter—light source (LS) and a photo detector (PD). Most commonly, the LS element is formed by one or more light-emitting diodes (LEDs). The PDs usually contain photodiodes, less commonly phototransistors or photoresistors. In the transmission mode, the LS and PD are placed on two opposite sides of the measured human tissue, for example, on a finger or an earlobe. In the reflection mode, the LS and PD measuring the intensity of the reflected and backscattered light are placed on the same side of the body surface and practically any skin area can be used here. The transmission PPG probes usually have a form of a finger ring (FR), a finger clip (FC), or an ear clip (EC)—see documentary photos in Figure 2a–c. The finger types are mostly used in medical applications—the FC is useful for pulse oximetry, as its clip exerts a pressure sufficient to eliminate the venous component in the tissue under the probe [17]. The EC realization is inconvenient for long-term measurement and recording but it is preferred in heart rate monitoring in situations when hands of the examined person cannot remain at rest [18].

The reflectance sensors, offering higher flexibility for PPG signal measurement from different locations on the body, are more suitable for the non-invasive wearable long-time monitoring devices. This type of PPG sensor is the most universal with practical placement mainly on fingers (typically fixed on a finger by an elastic/textile ribbon), as documented in Figure 2d, and on a wrist as various multi-functional smart watches or fitness bracelets. High-priced devices have three pieces of LEDs arranged in a triangle and multiple (three or more) sensing PHDs, as documented by a photo in Figure 2e. In addition, the PPG signal, together with the determined HR values, can be transmitted via the wireless connection to an external device (smartphone, tablet, etc.) for storage or next processing. On the other hand, there exist also specialized reflectance in-ear PPG sensors (placed in the auditory canal) which must be individually customized depending on the ear channel proportions [19]. These types of PPG sensors, mainly used in the systems for SpO_2_ continual monitoring, are usually connected to the POXI device by an electric cord [20].

The picked-up PPG signal has a typical amplitude modulation (ripple) with a partially linear trend (LT). A de-trending operation [15] is important in the case of a raw PPG signal, but it is usually not applied on VPG and APG signals. In addition, the PPG signal generally contains a noise component and can be often partially disturbed or degraded [19]. Therefore, the sensed PPG signal must be filtered prior to its processing and determination of HR values. A smoothing approach suitable for real-time processing of this relatively slow signal is a moving average (MA) filter with rectangular weighting. This basic smoothing method working in the time domain is used to filter out high frequency fluctuations in the PPG signal [24]. The proper choice of the MA window length is important—too long a window causes unacceptable decrease in the signal amplitude and loss of details, whilst a very short window does not deliver the required filtering effect. Analysis of MA filtering impact on the PPG signal properties was already analyzed in our previous work [12]. From our practical experiments, it follows that the *N*x parameter representing the half length of the MA window should be selected in the interval of 4–12 samples (if the sampling frequency of 125 Hz is applied).

For an analysis of morphological features of individual PPG pulse waves, a relatively high sampling frequency *f*_S_ (higher than 500 Hz) is required [25]. On the other hand, in continuous long-term screening and monitoring, sampling at lower frequencies may be used, for example, 128 Hz in a wrist-based wearable device for atrial fibrillation detection [26]. Commercial, wearable PPG sensors use typical sampling frequencies between 50 and 100 Hz [3]. For the purpose of this study, we determined and analyzed the energetic, time, and statistical parameters of the sensed PPG waves representing signal properties together with heart rate values. Using different *f*_S_ from the investigated range cannot change the subsequently detected pulse period and the finally determined HR; only the precision of the systolic and diastolic peaks decreases for lower *f*_S_.

### 2.2. Determination of PPG Signal Properties and Heart Rate Calculation

In the frame of off-line post-processing and analysis of the PPG wave, the signal envelope can be determined by low pass filtering of the squared input signal. In our case, the upper and lower envelopes (*E*_HI_, *E*_LOW_) are determined by the root-mean-square (RMS) method using a sliding window. The basic energetic parameters: amplitude, signal range, and signal modulation, are determined next. These parameters are subsequently used in the heart rate calculation process. For calculation of the mean heart peak amplitude (*HP*_AMPL_), the maximum (*Lp*_MAX_) and minimum (*Lp*_MIN_) levels of these peaks must be determined together with the offset level of the PPG signal (*L*_OFS_). The absolute *HP*_AMPL_ is calculated as:

(1) $$HP_{\mathrm{AMPL}}=(Lp_{\mathrm{MAX}}+Lp_{\mathrm{MIN}})/2-\bar{L_{\mathrm{OFS}}},$$

where the $\bar{L_{\mathrm{OFS}}}$ is the mean signal offset value.

Then, the relative heart peak ripple *HP*_RIPP_ of a PPG wave is calculated using *Lp*_MAX_ and *Lp*_MIN_ level values previously determined:

*HP*_RIPP_ = (*Lp*_MAX_ − *Lp*_MIN_)/*Lp*_MAX_ × 100 [%]. (2)

Finally, the relative PPG signal range (*PPG*_RANGE_) can be determined from the mean heart peak amplitude in percentage as:

*PPG*_RANGE_ = *HP*_AMPL_/*AD*_RES_ × 100 [%], (3)

where the *AD*_RES_ parameter depends on the resolution of the currently used A/D converter for digitalization of the analogue signal from the optical sensor and practically represents an actual numerical range of the processed PPG signal.

**Figure 3:**
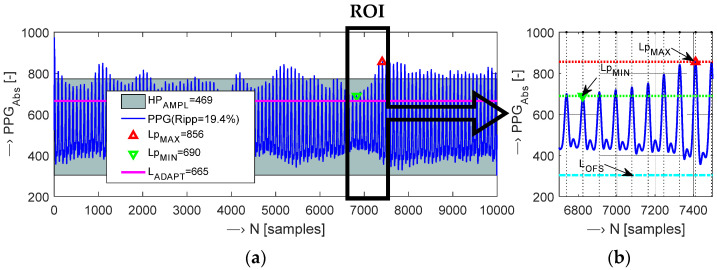
Visualization of adaptive signal level threshold determination: (**a**) whole 10-k sample PPG signal with determined *Lp*_MAX_, *Lp*_MIN_ levels, *HP*_AMPL_ and heart ripple parameters, and finally calculated *L*_ADAPT_ level, (**b**) ROI within 80th–89th PPG cycles with localized systolic peaks, determined *Lp*_MAX_/*Lp*_MIN_ values, and global *L*_OFS_ value; *f*_s_ = 100 Hz.

The HR determination algorithm must be simple and fast to be universally used in real-time processing of a continually sensed PPG signal. The HR may be estimated from the PPG signal by various methods [5,19,27]. The specialty of our approach to HR determination lies in the processing of a positive-valued PPG signal with the range of 0 to *AD*_RES_ instead of a signal of both polarities within the range from −1 to 1. The currently proposed algorithm determines HR values from the picked-up PPG signal in the following way: (1) the PPG signal threshold *L*_THRESH_ is set; (2) the pulse wave is clipped binary; (3) the heart pulse periods *T*_HP_ are determined; (4) the HR values are calculated. The signal threshold *L*_THRESH_ is given by a fixed value *L*_BASIC_ or by an adaptive threshold *L*_ADAPT_. The *L*_BASIC_ threshold value should cross the lower third of the PPG signal range predicted according to the measured PPG signal parameters. The *L*_ADAPT_ threshold value is specified as smaller than the smallest peak of all of the heart systolic pulses, see visualization in a documentary example in Figure 3.

The clipping operation produces a sequence *c*_PPG_ (*n*) of values 1/0 corresponding to the input signal samples above/below *L*_THRESH_ as:

(4) $$c_{\mathrm{PPG}}\left(n\right)=\{\begin{array}{cc} 1 & y\left(n\right)\geq L_{\mathrm{TRESH}}\\ 0 & y\left(n\right)⊲L_{\mathrm{TRESH}} \end{array}\;1\;\leq \;n\;\leq \;M,$$

where *M* is a total number of processed samples of a PPG signal. The heart pulse periods *T*_HP_ in samples are determined from this clipped sequence as the length of two adjacent segments of ones *T*_1P_ and zeros *T*_0P_ (*T*_HP_ = *T*_1P_ + *T*_0P_). Using the sampling frequency *f*s, HR values are calculated as:

HR = 60 × *f*s/*T*_HP_ [min^−1^]. (5)

**Figure 4:**
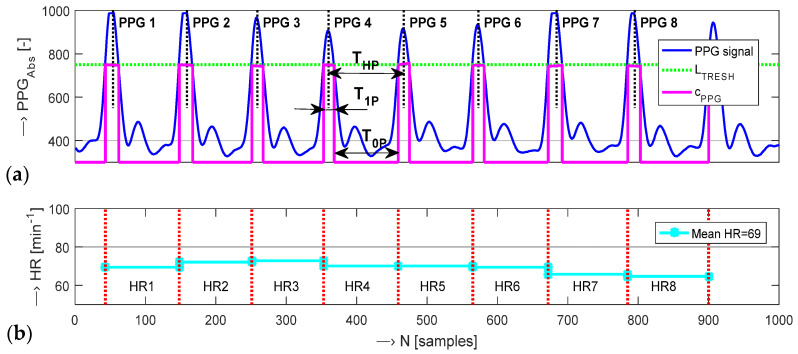
An example of HR determination: (**a**) 1k-samples of a PPG wave together with signed heart pulse periods *T*_HP_, lengths *T*_1P_, *T*_0P_, and a clipped sequence *c*_PPG_ (**b**) HR values corresponding to periods *T*_HP_ together with a final mean HR; *L*_THRESH_ = 750, *f*_s_ = 125 Hz.

The whole process of HR calculation is graphically demonstrated by an example in Figure 4.

To describe stability of the calculated HR values, a relative variability *HR*_VAR_ is calculated based on the mean HR and the standard deviation *HR*_STD_ as:

*HR*_VAR_ = (*HR*_STD_/*HR*_MEAN_) × 100 [%]. (6)

For specifying the accuracy of the determined HR values from the PPG signal (*HR*_PPG_) a comparison with those measured by blood pressure monitor (BPM) and/or oximeter devices (*HR*_CTRL_) is used. When the control HR measurement by a BPM device is applied, the *HR*_CTRL_ parameter represents one value *HR*_BPM_ obtained for the whole measuring interval with duration *T*_DUR_. In the case of using an oximeter which produces continual values of HR during the whole measurement, the *HR*_CTRL_ is defined as the mean value. In this way we obtain the differential parameter *HR*_DIFF_ relative to the mean *HR*_PPG_ ($\bar{HR_{\mathrm{PPG}}}$) in [%] defined as:

(7) $$HR_{\mathrm{DIFF}}=(HR_{\mathrm{CTRL}}-HR_{\mathrm{PPG}})/\bar{HR_{\mathrm{PPG}}}\;\times \;100\;[\%].$$

The acquired *HR*_DIFF_ values are statistically processed for final graphical comparison in the form of bar-graphs, boxplots, and histograms.

**Figure 5:**
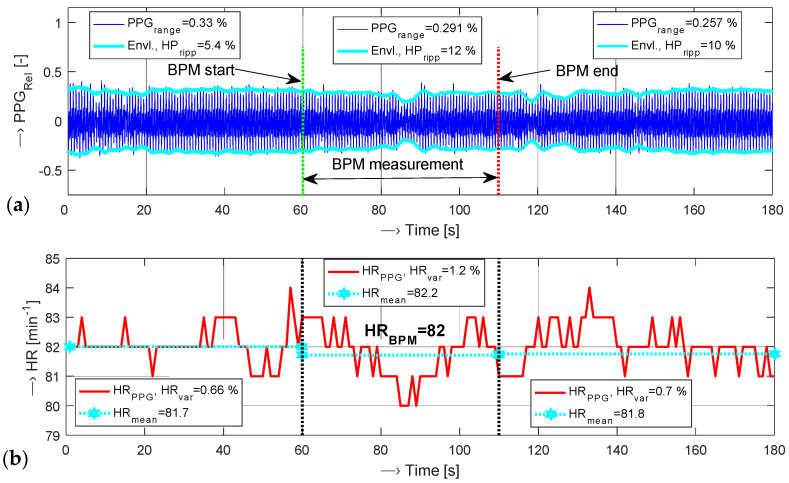
Visualization of a sensed PPG wave with parallel measurement by the BPM device on different arms in three phases (before, during, and after the BPM measurement): (**a**) sensed PPG wave with calculated signal range, envelopes, and ripple, (**b**) HR values from the PPG signal compared with *HR*_BPM_ together with *HR*_VAR_ and *HR*_MEAN_ values; a PPG sensor is worn on a left forefinger, a BPM device on a right arm.

The *HR*_DIFF_ parameters can only be determined by the steady PPG signal. Usually, the measurement by the BPM device and the PPG signal sensing is realized on the opposite arm/hand to minimize the pressure effect on the blood vessels in the arm. In this case, we observed a higher ripple of the PPG wave during the measurement time interval (between BPM start and BPM end) and a small decrease in the signal amplitude (range). This phenomenon has minimal influence on the finally determined HR values as shown in an example in Figure 5. If the arm for BP measurement and the hand for parallel PPG signal recording are the same, the recorded PPG cycles may be missing or having very low amplitude due to compressed blood vessels in the arm. For a detection of these events, we created the so called no-pulse signal function *HP*_NOS_ with the value of “1” for no HR pulses present in the PPG signal and the value of “0” otherwise. For practical calculation of this function, the envelope threshold *E*_THRESH_ based on the possible minimum upper and lower envelopes *E*_HI_, *E*_LOW_ must be determined from the whole processed PPG wave consisting of *P* determined PPG cycles. Then, each systolic pulse amplitude *Ap*_j_ is compared with the *E*_THRESH_ value:

(8) $$HP_{\mathrm{NOS}}=\{\begin{array}{cc} “1” & Ap_{j}\in \langle -E_{\mathrm{THRESH}},E_{\mathrm{TRESH}}\rangle \\ “0” & Ap_{j}>\left|E_{\mathrm{THRESH}}\right| \end{array}\;1\;\leq \;j\;\leq \;P.$$

**Figure 6:**
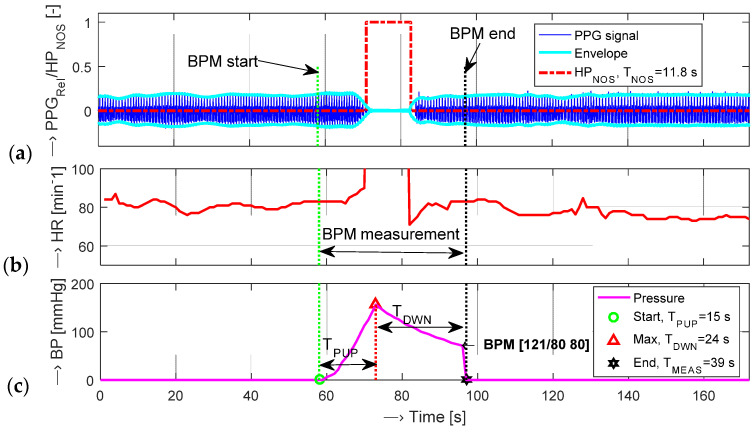
Visualization of a PPG wave sensed from the left little finger with parallel BP measurement on the same arm: (**a**) the PPG signal and its envelopes together with determined *T*_NOS_ ≅ 10 s, (**b**) HR values determined from the PPG signal, (**c**) the pressure characteristic measured by the BPM device with determined *T*_PUP_, *T*_PDWN_, and *T*_PMEAS_ time intervals, BP and HR values.

The final no-signal time duration *T*_NOS_ is calculated from the whole PPG signal with a total time duration *T*_DUR_ as a duration of the time interval in which the *HP*_NOS_ value is “1”–see the red dash-dotted line in Figure 6a. HR values detected from the PPG signal are shown in Figure 6b. For the time interval *T*_NOS_ when HP_NOS_ is equal to 1, the HR values are not defined, so the maximum possible value is substituted there (HRmax = 130 min^−1^). The simultaneously picked-up pressure characteristics of the tested BPM device is then used for determination of the following three temporal parameters: *T*_PUP_—time interval of increasing the air pressure in the measuring cuff, *T*_PDWN_—time interval of squeezing the air out of the cuff, and *T*_PMEAS_—time duration of the whole measuring process, as documented in Figure 6c.

## 3. Objects, Experiments, and Results

### 3.1. Principal Description of Developed Prototypes of PPG Sensors

The PPG sensor for real-time continuous PPG signal measurement principally consists of 4 functional blocks: (1) optical part with LS transmitter and PD receiver together with the analog interface for basic pre-processing of an electrical signal picked up by the PD, (2) microcontroller part containing an analog-to-digital (A/D) converter and a serial communication interface, (3) wireless communication part for receipt/transmission of commands/data from/to an external device, (4) power supply part. The sensor’s power supply can be realized by rechargeable battery cells or power banks to prevent any galvanic connection with other devices powered by a standard AC line of 230 V and 50 Hz (in Europe). To enable its operation in a low magnetic field, the sensor must be made of a non-ferromagnetic material including the power supply. In addition, due to a strong RF disturbance in the scanning area of the MRI device, all of the parts must be shielded.

**Figure 7:**
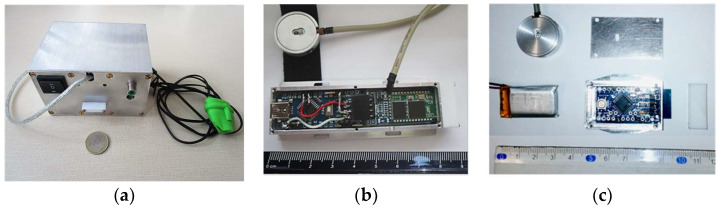
Assemblage with aluminum covering of: (**a**) prototype of the PPG-EP sensor, (**b**) the body and optical part of the PPG-PS1 sensor, (**c**) the body, optical part of the PPG-BLE sensor, and the Li-Po battery used for power supply.

The first prototype of the PPG signal sensing tool (further called “PPG-EP”) operating in the transmission mode is based on the optical sensor HRM-2511E by Kyoto Electronic Co., China, practically realized in the form of a rubber finger ring (see the photo in Figure 2a). The output from the PHD is pre-amplified and double filtered in a cascade connection using the Easy Pulse sensor v 1.1 module (ER-CDE10301E) by Embedded Lab, Williamsburg, VA, USA. Each of the 2 cascade blocks in this analogue interface consists of a passive RC high-pass filter (HPF) with the cut-off frequency of about 0.5 Hz and an active low-pass filter (LPF) with the cut-off frequency set to 3.4 Hz [20]. The output from the second LPF block is passed to the input of a 10-bit A/D converter integrated in the 8-bit processor ATmega328P by Atmel Company. This processor is a core element of the microprocessor unit—the Arduino Uno v. 3.0 board [28] together with an USB interface which can also be supplemented by the GSM module for connection to a mobile data gateway [29]. For our purpose, the BT communication module HC-06 [30] is used. It supports the bi-directional data transfer in BT 2.0 standard at 2.4 GHz with the maximum baud rate of 115,200 bps. All 3 parts are powered via the USB port by 5 V power bank AlzaPower Source 20,000 Quick Charge 3.0 by Alza.cz—see the overview assembled photo in Figure 7a. Due to the described type of the analogue interface, this PPG sensor produces the second derivative of the PPG wave in the final effect (compare PPG wave types in Figure 1).

The next two prototypes of the PPG sensor are based on the optical Pulse Sensor Amped (Adafruit 1093) [31] working in a reflectance mode and having an analogue interface integrated on board with a light transmitter and receiver. The integrated analogue interface consists of one step pre-amplifier, passive high-pass/active low-pass signal filters with the same cut-off frequencies as Easy Pulse module, so the finally produced PPG wave represents the first derivative of the originally sensed PPG signal. These two prototype realizations differ in the used microcontroller board, the BT communication module, and the power supply method—see Table 1 and Table 2. 

The second compared sensor in this study (further called “PPG-PS1”) was developed using the Arduino Nano v. 3.0 board [32], which is also based on the processor ATmega328P working with the clock frequency *f*_CLK_ of 16 MHz, and an USB interface. For communication with the control device, the sensor is connected to the mentioned BT module HC-06 (see photo in Figure 7b). All 3 components are powered via the USB port by a cable from an external 5 V power bank THAZER.

The last developed PPG sensor (further called “PPG-BLE”) is based on Arduino Pro Mini v. 2.0 board [33] also with the processor ATmega328 but without the USB interface and running at *f*_CLK_ = 8 MHz. For serial communication, the BT module MLT-BT05 by Techonics Ltd. (Lahore, Pakistan) [34] working in the BT4.1 BLE standard was applied. The 3.7 V rechargeable polymer-lithium-ion (Li-Po) cell was used for sensor powering. The battery is practically mounted directly on the top of the shielding aluminum box. The applied assemblage and realized shielding by aluminum boxes for both prototypes of PPG sensors working in a reflectance mode are documented in photos in Figure 7c.

In summary, it can be specified that all of the 3 investigated PPG sensor realizations:

1. have a common basic structure consisting of four functional blocks;
2. use Arduino boards based on the processor ATmega328P with integrated 10-bit A/D converters, so the theoretical maximum *AD*_RES_ value is 2^10^ = 1024;
3. enable real-time PPG signal sensing in the low magnetic field with RF disturbance environment;
4. the sensor’s body and optical part are covered by aluminum boxes;
5. work in the slave mode: after initialization they wait for commands from the master device via the BT connection;
6. PPG signal sensing in 2 operating modes: (1) PPG wave pick up in data blocks with the length of *N*_MEAS_ = {1k, 4k, 10k, and 25k} using 16-bit binary data samples; (2) continual PPG signal measurement that begins and ends by <Start> and <Stop> commands;
7. work with the control application “PPGsens7BT.exe” developed for Windows platform (successfully tested under XP, 7, and 10 versions) created for the master device to control the PPG sensor;
8. a service program implemented in the microcontroller board supports measurement and data transmission;
9. adjustable time delay to read the analog signal from the optical sensor, to perform 10-bit A/D conversion and data transmission to the control device;
10. transmission of data blocks with the length of *N*_MEAS_ = 1k-samples are used for monitoring and display of the currently sensed PPG signal. For practical PPG signal measurement, the setting of *N*_MEAS_ = 10k or 24k-samples is usually applied enabling a 1-shot storage of the PPG wave with duration of 80 or 240 s (using *f*_S_ = 125 Hz). If longer time durations of sensed PPG signals are required, the signal data can be stored directly to an output wave file. Other requested time durations of sensed PPG signals can be solved using the direct storage to an output wave file.

On the other hand, Table 1 summarizes differences in an architecture, basic components, and mechanical realizations, and Table 2 shows detailed differences in electrical and functional parameters of all three PPG sensors.

### 3.2. Description of the Control Application Based on Windows Platform

The control application *PPGsens7BT.exe* was created for the master device to control the whole process of the PPG signal acquisition and post-processing consisting of: (1) real-time monitoring and displaying of the PPG signal, (2) continuous PPG signal measurement for the selected sampling frequency *f*_S_ and the post-processing operations. This second operation mode enables: (a) direct saving of the unprocessed PPG data to a file on the hard disk of the control device, (b) saving of the acquired PPG data to an internal memory buffer for further processing such as filtering and HR determination; in this way the modified data can be stored off-line to the file(s) by an application user. In this case, a fixed number of samples *N*_MEAS_ is automatically transmitted from the PPG sensor to the control device. The received PPG signal is stored in a Wave format (with 16-bit quantization, mono, PCM coding), so the signal must be first converted to the bipolar relative representation (in the range from −1 to 1). The stored PPG signal records can be further processed and analyzed off-line in the Matlab program environment.

**Figure 8:**
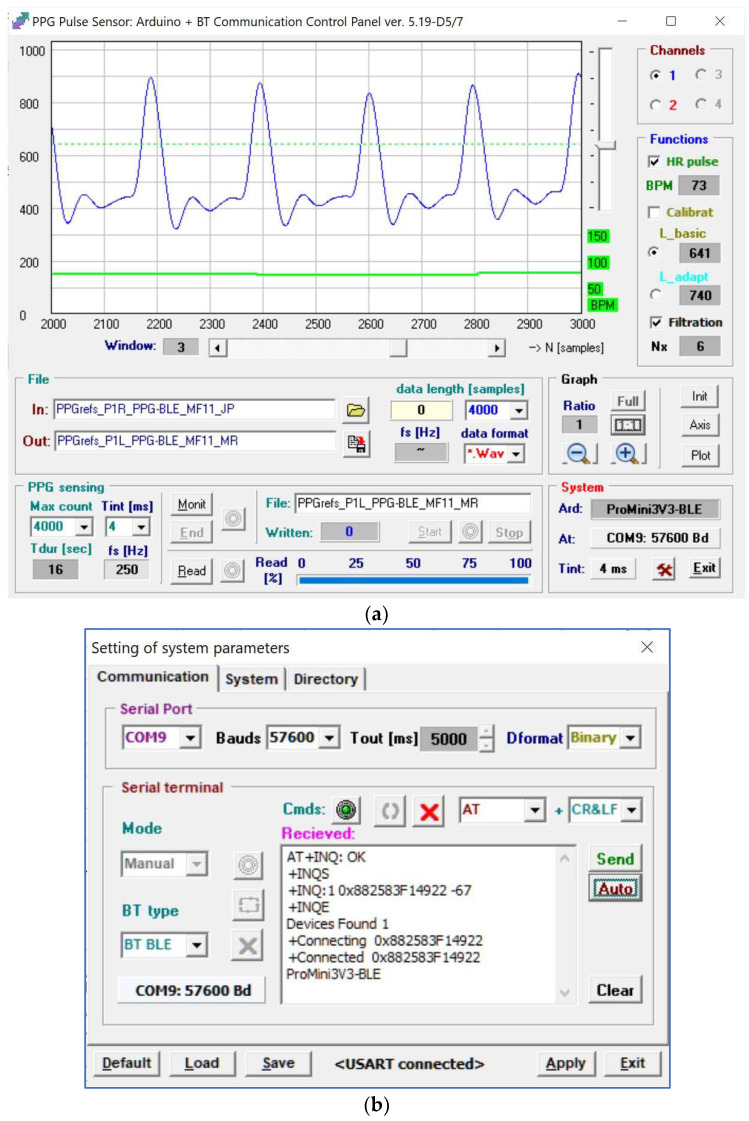
Screen copy of the control application *PPGsens7BT* v.5.19: (**a**) main operating window with a displayed PPG wave (4th frame of 4-k sample data block, *f*_S_ = 250 Hz) and a determined mean HR value in beats per minute, (**b**) the parameter setting window documenting the process of BT connection creation with the *PPG-BLE* sensor based on the BT module MLT-BT05.

The developed application enables communication with a PPG sensor in three modes: (1) “off-line”: without any BT communication—only the stored PPG waves can be shown, analyzed, and processed; (2) “automatic”: with automatic connection to a pre-defined type of an external PPG sensor (by initial setting of program parameters stored in *.INI file)—real-time PPG signal monitoring as well as continual measurement including subsequent storing of received data is enabled; (3) “manual” mode beginning with the application start without BT connection, proceeding with a manually established connection to a chosen type of a PPG sensor and communication parameters (serial channel type, baud rate, BT 2.0/BT 4.1 BLE protocol, etc.) working similar to the automatic mode. In addition, within this mode it is also possible to perform manual disconnection and/or again to create a connection with another PPG sensor using different parameters. An example of a screen copy of the main control window of the application *PPGsens7BT* (version 5.19) is shown in Figure 8a. The activated setting windows with showed listing during establishing of the BT connection with the PPG sensor working at the standard 4.1 BLE—transmitted and received AT commands for MLT-BT05 module can be seen in Figure 8b.

### 3.3. Performed Measurements and Analyses

The main aim of all of the performed measurements and analyses was to find suitable and the most universal setting of parameters for PPG signal real-time recording as well as off-line post-processing in different working mode conditions including proper function in a low magnetic field environment. The comparison of three prototypes of the developed PPG sensors was realized in four phases:

1. Preliminary testing and verification of the functionality of the PPG sensors in cooperation with the control application for three operation modes: (1) without BT connection (NC), (2) after established connection to the control device (CE), (3) during real-time transmission of PPG signal samples to the control master device (MC). This phase was accompanied by a measurement of the sensor’s mean DC using a different power supply of 5/3.7 V in the 3 mentioned operation modes;
2. Testing of the precision and stability of the HR values determined from the PPG signal by comparative measurement with another commercial HR measurement device (portable BPM) and by a POXI device, all in the normal laboratory conditions. In addition, a supplementary analysis of the influence of the BP/HR parallel measurement by a BPM device on the same hand as the PPG signal recording was performed;
3. Analyzing the functionality, quality, and stability of the BT connection between the tested PPG sensor located inside the scanning area of the MRI device and the control device located outside the shielding cage. Additional testing of the influence of the BT transmission in the scanning area on the quality of the finally obtained MR images;
4. First-step measurement of the PPG signals in a low magnetic field environment of the MRI device, analysis of the PPG signal properties.

In the third and fourth phases, the experiments were performed under three different conditions: (1) the door of the shielding cage open (OD) and the MRI device executing no scan sequence (NS), (2) the door of the cage closed (CD) and NS, (3) CD and a MR scan sequence is running (SR). Testing of the quality of obtained MR images was only realized in the SR condition.

### 3.4. Experimental Conditions

**Figure 9:**
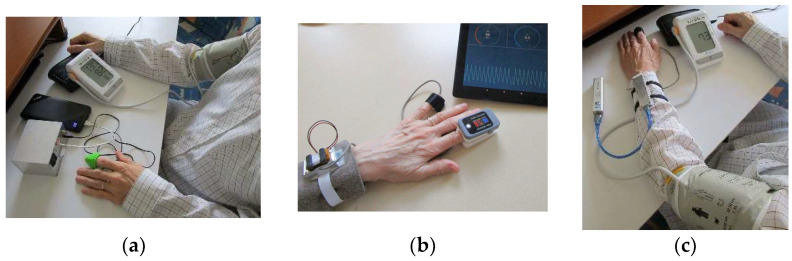
Arrangements of comparative measurements of HR values from the PPG wave and by the BPM and POXI devices: (**a**) with parallel measurement by the BPM device on the right hand and the *PPG-EP* sensor on a forefinger of the left hand; (**b**) parallel sensing of the PPG signal via the POXI with BT data transfer to a tablet and by the *PPG-BLE* sensor using a forefinger and a pinkie of the left hand; (**c**) parallel measurement by the BPM device and by the *PPG-PS1* sensor worn on a forefinger-both placed on the left hand.

In the frame of our previous research [10,11], we have also compared results of HR values measured by three tested BPMs with those determined from the PPG signal. The best results with minimal dispersion and approximately zero mean value of calculated relative differences were achieved by the automatic blood pressure monitor BP A150-30 AFIB by Microlife AG, Swiss Corporation, Widnau/Switzerland. Hence, this type of BPM was used in the present experiments [35]. To prevent the already discussed possible negative influence of an inflated pressure cuff of BPM on a tested person’s blood system, the PPG signal was picked up from a forefinger of the opposite hand—sees an arrangement photo in Figure 9a. In the second type of comparative measurement experiments, the PPG signal was sensed and measured in parallel for calibration of the determined HR values with the help of the oximeter Berry BM1000C [36] by Shanghai Berry Electronic Tech Co., Ltd., Shanghai, China. This POXI device works in a transmission mode and enables also recording of the blood oxygen saturation and transfer of the HR values to the control device (tablet) via BT connection. PPG sensors were successively attached to little fingers of both hands by an elastic ribbon, the POXI device on the forefingers as documented in Figure 9b. The recording of PPG signals as well as oximeter values lasted for 80 s. The HR values obtained in this way were subsequently processed off-line and analyzed statistically. In the currently realized comparative measurements two female and six male healthy volunteer persons (authors themselves and their colleagues with average age of 50 years) were joined.

In the supplementary experiments with a parallel measurement by a BPM device and a PPG sensor placed on the same hand, the PPG sensor was worn on a forefinger—a practically used arrangement is shown in a photo in Figure 9c.

The third and fourth phase of the experiments were realized in a low magnetic field environment of the open-air MRI device E-scan Opera by Esaote S.p.A. [13] that is located at the Institute of Measurement Science, Slovak Academy of Sciences in Bratislava. The static field with magnetic induction of 0.178 T is formed between two parallel permanent magnets of this MRI scanner. It is placed in a metal cage to suppress high-frequency interference. This cage is made of a 2-mm thick steel plate with evenly spaced holes of 2.5-mm diameter in a 5-mm grid to eliminate the propagation of the electromagnetic field to the surrounding space of the control room [13].

**Figure 10:**
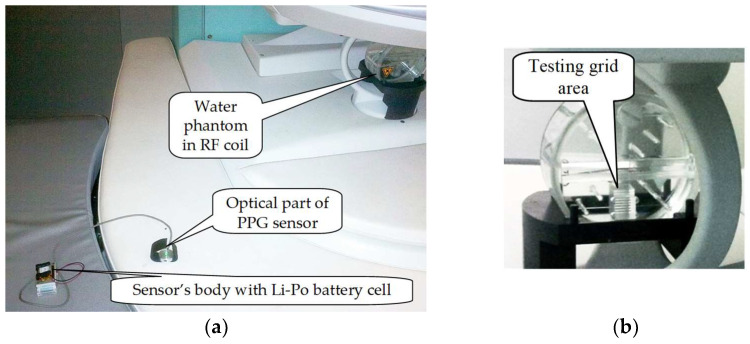
Arrangement of RSSI parameter measurement inside the MRI device E-Scan Opera: (**a**) overall photo with currently tested *PPG-BLE* sensor, (**b**) detail of used water phantom with a special testing grid area inside the RF receiving/transmitting coil.

In the preliminary experiments, PPG sensors were located inside the scanning area of the MRI device and no testing person was present; only a testing water phantom with a special testing grid area (TGA) was placed in the RF coil for generating MR images. The PPG sensor’s body, together with its optical part, were laid on a patient’s bed (see photos in Figure 10). The quality of BT connection was evaluated by the Received Signal Strength Indicator (RSSI) parameter representing an estimated measure of power level that an RF client device receives from an access point [37]. At larger distances, the signal becomes weaker and the wireless data rates become slower, leading to a lower overall data throughput. Measurement of the RSSI parameter was performed in three mentioned operating conditions {OD&NS, CD&NS, CD&SR}; during measurement in the CD&SR condition, the Hi-res SE-HF scan sequence with TE = 26 ms, TR = 500 ms, and sagittal orientation was executed. The distance between the PPG sensor and the control device was *D*x = 225 cm, both connected devices were in the approximate height of 75 cm from the floor. For each of the sensor prototypes in every condition, the RSSI value was measured three times in [dBm] during the BT connection establishment (see the documentary screen copy of the communication setting window of the *PPGsens7BT* application in Figure 8b). For usage in final comparison, the mean value was determined from these three obtained values. The same experimental arrangement was used in the investigation of the influence of BT transmission on the quality of MR images. Only the *PPG-EP* prototype in the SR condition was tested in this investigation because it consists of the BT module HC-06 working with higher levels of the communication signal energy than the BT module MLT-BT05 operating in the BT 4.1 BLE standard. Therefore, we suppose that the assumed RF disturbance will be principally higher than in the case of the prototype *PPG-BLE*.

**Figure 11:**
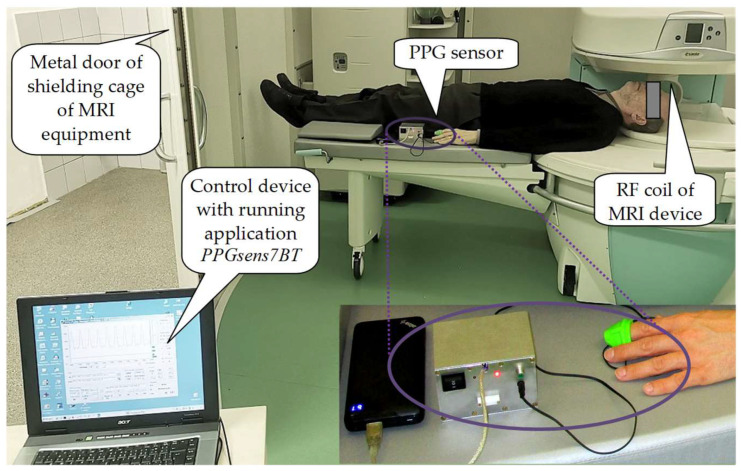
Measurement of a person lying in the MRI device Opera using the *PPG-EP* sensor with BT data transmission to a control device located outside the shielding metal cage.

In the first PPG signal measurement, the testing person was lying on a patient’s bed with a head placed near the RF sensing coil in the middle of the scanning area (see the arrangement photo in Figure 11). For testing of the sensor *PPG-EP* operating in the transmission mode, the rubber finger ring with optical part of the sensor was put on the left/right hand forefingers. The sensor’s body and the supply power bank were loosely laid on a patient’s bed near the left leg. In the case of testing of the sensor prototypes working in the reflectance mode (*PPG-PS1* and *PPG-BLE*), the PPG sensor body with a power supply source was mounted on the left/right hand’s wrist and the optical part of the sensor was fixed on a forefinger by an elastic ribbon. Within the CD&SR measuring condition, the scan sequence 3D-CE (with TE = 30 ms, TR = 40 ms; 3D phases = 8) was running. In this way, six PPG signal records per a tested person were formed. In the frame of this measurement, small databases of PPG signals from eight healthy volunteers were collected and further processed. The examined persons were the authors themselves and their colleagues in the age between 20 and 59 years—4 females and 4 males.

## 4. Discussion of Obtained Results

The preliminary experiments in the standard laboratory conditions confirm the practical functionality of all three tested realizations of PPG sensors with real-time BT data transfer mastered from the control application running on the laptop with Windows 10 OS. Due to the required real-time function of all 3 investigated PPG sensor prototypes, the recommended sampling frequencies were *f*_S_ = {100, 125, 200, 250, and 500 Hz}. On the other hand, sensing of the PPG signal with *f*_S_ higher than 250 Hz is only reasonable in the case of the systolic pulse width determination with higher accuracy. In our case the precise shape of peaks is not so relevant; rather, only the detected heart pulse period is necessary for correct HR value calculation. Therefore, the setting of *f*_S_ = 125 Hz was finally chosen for use in further measurement experiments. A measurement of the sensor’s mean DC shows relatively great independency on the used operating voltage, type of a power supply, and a microcontroller board—see the values in Table 3. Chosen types and practically used power banks and Li-Po battery cells with their capacity guarantee safe power supplying for long-term PPG signal sensing experiments.

**Figure 12:**
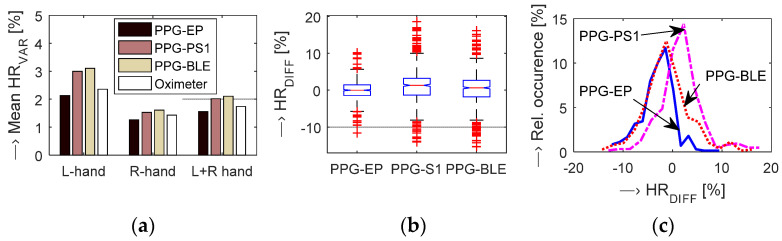
Statistical results of comparative measurements by the oximeter device for all three tested PPG sensor prototypes: (**a**) bar-graph of mean variances of HR values separately for both hands, (**b**) boxplot of basic statistical parameters of *HR*_DIFF_ values joint for both hands, (**c**) histograms of *HR*_DIFF_ values for left and right hands together; all tested persons.

In the frame of the second phase experiments, the accuracy and stability (fluctuation) of the heart rate determined from the PPG signal was compared with discrete values obtained by the BPM device as well as with continual values transmitted from the oximeter. Statistical results of comparative measurements by the OXI device shown in Figure 12 demonstrate the lowest *HR*_VAR_ values for the *PPG-EP* sensor and the highest ones in the case of the *PPG-PS1* prototype. HR fluctuation of the used OXI device was comparable with the *PPG-EP* sensor but it was lower than the values obtained by PPG sensors working on a reflectance principle. Essential differences were detected according to the used hand (PPG signal sensed on the left hand had higher variation than from the right hand) but not according to the gender of a tested person (male vs. female). These results are in principal correspondence with others obtained by comparative measurements with the help of the BPM device as documented by numerical results in Table 4. However, HR stability parameters obtained from parallel measurements by the OXI device have shown greater *HR*_VAR_ and *HR*_DIFF_ values. Both parallel measurements have shown that the *PPG-PS1* realization enables HR determination with positive *HR*_DIFF_ variation of the measured values while the *PPG-EP* and *PPG-BLE* prototypes give negative *HR*_DIFF_ variation as can be seen on the histogram in Figure 12c and in the right part of Table 4.

**Figure 13:**
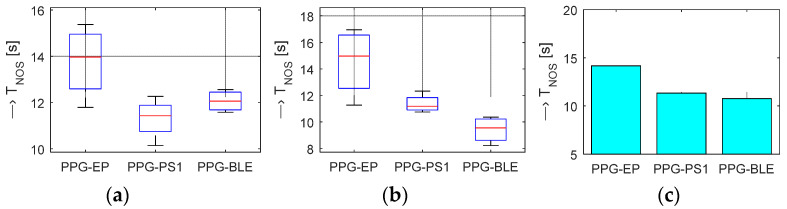
Statistical comparison of *T*_NOS_ time intervals for tested PPG sensors: (**a**) boxplot of *T*_NOS_ values for left hand forefingers, (**b**) boxplot for right hand forefingers, (**c**) mean *T*_NOS_ values for both hands together; all tested persons.

The additionally obtained experimental results confirm our assumption that the parallel measurement by the BPM device on the same arm has significant negative influence on the PPG signal sensed from the fingers which is manifested by generation of a no-signal time interval with the duration *T*_NOS_. In the final effect, differences were observed in blood circulation in the measured arm. Analysis of the determined *T*_NOS_ values shows that time intervals are the longest for the *PPG-EP* prototype with an optical sensor working in the transmittance mode. Shorter *T*_NOS_ intervals were observed in the case of prototypes using the reflectance optical sensor but there are no significant differences between *PPG-PS1* and *PPG-BLE* prototypes. In contrast, all three prototypes differ in *T*_NOS_ values depending on the hand type—see statistical results for all tested persons in Figure 13.

Preliminary mapping of conditions has confirmed the possibility of functional wireless BT connection and data transfer through the shielding cage of the tested MRI device with lower signal amplitude. It is well documented by the obtained RSSI values for three tested experimental conditions provided in Table 5. The minimum RSSI of −94 dBm was reached for the *PPG-BLE* sensor realization when the MR scan sequence was running, but also in this case the BT connection was stable, and the received PPG signal was usable (without any disturbance or artifacts). An investigation about the influence of the BT transmission inside the scanning area of the running MRI device on the scanning process confirms our working presumption.

**Figure 14:**
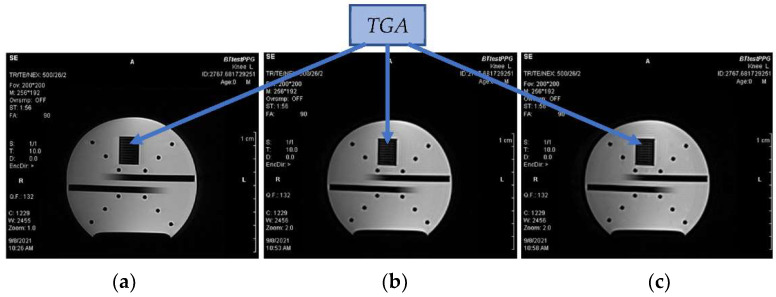
MR images of the water phantom with the testing grid area obtained in three BT communication conditions of the *PPG-EP* sensor with the HC-06 BT module placed in the scanning area of the MRI device: (**a**) without any BT connection, (**b**) after established connection to the control device, (**c**) during data transmission to the control master device; executed Hi-res SE-HF scan sequences with TE = 26 ms, TR = 500 ms, and sagittal orientation.

For an analysis of the influence of BT transmission during the scanning process of the running MRI device on the quality of the obtained MR images, only the *PPG-EP* prototype with HC-06 BT module was tested. The water phantom presented in Figure 10b was used for this testing. The obtained results in the form of MR images of this water phantom rotated by 180 degrees can be seen in Figure 14. The achieved quality factors of the images are the same (Q.F. = 132), but careful observation of these images reveals soft horizontal lines inside the phantom’s special grid area that are more visible in Figure 14b,c than in Figure 14a. Finally, we can generalize that the application of PPG sensing has a minimal influence on the quality of the obtained MR images.

**Figure 15:**
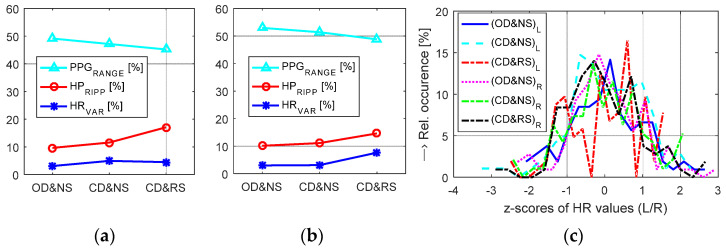
Comparison of PPG parameters using the *PPG-EP* prototype for three conditions inside the MRI device—both hands separately: (**a**) mean values of *PPG*_RANGE_, *HP*_RIPP_, and *HR*_VAR_ parameters for the left hand (**b**) the right hand, (**c**) histograms of z-scores calculated from HR values; PPG sensor was worn on the index fingers of L/R hands of a male.

First-step measuring experiments confirm practical usability of all three proposed sensors for long-term sensing of the PPG signal in the magnetic field environment with additional RF and EM disturbance. To guarantee secure serial BT communication between the PPG sensor and the control device through the shielding cage the baud rate must be decreased, especially in the case of the *PPG-BLE* realization (max. at 57,600 bps—see the fourth column in Table 2). The numerical comparison of PPG signal properties and HR parameters in Table 6 shows that the running scan process (CD&RS) has practically minimum influence on the sensed PPG signal. Higher *HR*_VAR_ values can be observed in the case of a group of female persons whose stress factor due to scanning inside the MRI device was probably greater. All 3 tested PPG sensors produce relatively stable PPG signals with the ripple up to 20%; the highest *HR*_VAR_ values were detected for the *PPG-PS1* prototype, the minimum values in the case of the *PPG-EP* sensor. The results of a detailed analysis of properties of the PPG signal sensed from a male person with the help of the *PPG-EP* prototype in three sensing conditions {OD&NS, CD&NS, CD&RS} are presented graphically in Figure 15a,b. The obtained results are principally in correspondence with the summary ones for {CD&NS/CD&RS} states. A decrease in a PPG signal range and an increase in *HP*_RIPP_, and *HR*_VAR_ parameters can be caused by a progressive stress effect on a tested person. The calculated histograms of z-scores of HR values for both hands show no significant differences in three sensing conditions. Nevertheless, histograms for the PPG signal sensed in OD&NS condition contain curves that are smoothed more than for CD&RS condition as documented in Figure 15c.

## 5. Conclusions

Investigated PPG sensor prototypes were developed especially for measurement in the magnetic field environment. The comparative measurements performed in laboratory conditions using the BPM and OXI devices show that all three tested realizations of PPG sensors have acceptable and similar accuracy of HR determination. The detailed statistical analysis provides the highest variance of *HR*_DIFF_ values in PPG signals sensed by the *PPG-PS1* type. The *PPG-EP* prototype with the optical sensor part working in a transmittance mode produces the smallest variance. There are essential differences between *HR*_VAR_ values determined from PPG signals of the left and right hands as presented by the graphs in Figure 12 and by numerical results in Table 4. The supplementary analysis has shown that the cuff must be placed on the opposite arm for proper BP measurement while the pressure effect on the PPG signal was practically none.

Additional analysis based on application of a special water phantom confirms our presumption that BT transmission in the scanning area of the running MRI device has practically no influence on the quality of the obtained scanner images. Finally, the performed measurements using testing persons lying inside the MRI tomograph during the scanning process have verified proper function of all three PPG sensors in the magnetic field with RF and EM disturbance and usability of the sensed PPG signals for further processing and analysis.

The *PPG-BLE* prototype with lower supply voltage produces a PPG signal with lower *PPG*_RANGE_ and subsequently worse signal-to-noise ratio. On the other hand, the acquisition should be battery saving to enable long-term PPG signal recording. Therefore, this requirement is fulfilled by using the low energy *PPG-BLE* sensor—as shown by the PPG sensors’ mean DC values in Table 3.

The main limitation of this study lies in the fact that only a small group of tested persons participated in the measurement of PPG signals due to a bad pandemic situation in our country and limited possibilities for experiment realizations, so these results cannot be generalized. Only healthy vaccinated people could participate (authors themselves and their colleagues from IMS SAS) for collecting the PPG signal databases. The second limitation lies in the fact that our open-air MRI device was devised for standard medical practice, but our institute does not hold a certificate for real patient examination, so it can only be used for non-clinical and non-medical research.

We plan to realize more measurement experiments inside the MRI device in the near future. Properties of the PPG signals picked up from fingers and wrists will be compared with HR values measured by examined persons’ smart watches and fitness bracelets. The whole-body MRI device TMR-96 also located at our institute will be used for PPG sensor prototype testing. In this MRI scanner with magnetic inductance of 0.1 T, the gradient system is formed by solenoid coils flown by stronger currents resulting in stronger electromagnetic disturbance. In the case of possibly using of PPG sensors in MRI devices working with strong magnetic induction up 3 T, we will require further structural and material modifications of both the optical part of the PPG sensor and its body. The aforementioned limitation of using only non-ferromagnetic parts and components must be strictly retained, because even small metal pieces can cause damage of the whole device or bring about any injury of a tested person. Finally, practically applicable power supply methods for PPG sensors have not yet been analyzed; here, the possibility of external power supply using a power-bank is excluded.

## Figures and Tables

**Table 1:** Differences in an architecture, basic components, and mechanical realizations of all three PPG sensor prototypes.

Sensor Prototype	Optical Sensor Part	Mounting Method	Wearable	Arduino Board ^(1)^	BT Module/Standard	Sensor’s Body Dimensions (L × W × H)	Sensor’s Body Weight
*PPG-EP*	HRM-2511E	rubber ring	No	Uno v. 3.0	HC-06/BT 2.0	105 × 70 × 80 mm	445 g
*PPG-PS1*	Adafruit 1093	1” aluminum target fixed by elastic ribbon	Yes	Nano v. 3.0	HC-06/BT 2.0	80 × 20 × 10 mm	38 g
*PPG-BLE*	Adafruit 1093		Yes	Pro Mini v. 2.0	MLT-BT05/BT4.1 BLE	40 × 25 × 15 mm	40 g ^(2)^

^(1)^ All Arduino boards are based on the processor ATmega328 with 10-bit A/D converters; ^(2)^ Including mounted Li-Po battery cell.

**Table 2:** Differences in electrical and functional parameters for three tested PPG sensors.

Sensor Prototype	Sensor Working Mode	Processor *f*_CLK_	Max. BT Baud Rate	Supported *f*_S_ for PPG Signals	Power Supply (Voltage/Capacity)
*PPG-EP*	transmittance	16 MHz	115,200 bps	{100, 125, 200, 250, 500, 1000} Hz	5 V/22,000 mAh
*PPG-PS1*	reflectance	16 MHz	115,200 bps	{100, 125, 200, 250, 500, 1000} Hz	5 V/2200 mAh
*PPG-BLE*	reflectance	8 MHz	57,600 bps	{100, 125, 200, 250, 500} Hz	3.7 V/125 mAh

**Table 3:** Comparison of PPG sensors’ mean DC values for three functional states and used power supplies.

Sensor Type	Supplying Method	Functional State		
		NC	CE	MC
*PPG-EP*	5 V Power bank via USB	53 mA	60 mA	68 mA
*PPG-PS1*	5 V Power bank via USB	18 mA	26 mA	30 mA
*PPG-BLE*	3.7 V Li-Po battery cell	13.5 mA	14 mA	17 mA

**Table 4:** Mean HR stability parameters calculated from the PPG signal measured in parallel by the BPM device for all tested persons.

Sensor ^(A)^/Parameters	*HR*_VAR_ [%]			*HR*_DIFF_ [%]		
	Left	Right	Both	Left	Right	Both
***PPG-EP***	3.16	2.66	2.91	−1.48	−1.24	−1.36
***PPG-PS1***	4.35	3.78	4.06	2.52	1.89	2.21
***PPG-BLE***	3.76	3.61	3.68	−1.82	−1.16	−1.49

^(A)^ Tested PPG sensors worn on forefingers of left/right hand, BPM device on an opposite arm.

**Table 5:** Mean RSSI values joined for two BT communication modules measured in three experimental conditions.

BT Module ^(A)^/Condition	OD&NS	CD&NS	CD&RS
HC-06 (5 V, BT 2.0)	−60 dBm	−90 dBm	−91 dBm
MLT-BT05 (3.3 V, BT 4.1)	−86 dBm	−92 dBm	−94 dBm

^(A)^ *PPG-EP* and *PPG-PS1* prototypes consist of the same BT module HC-06; *PPG-BLE* includes the MLT-BT05 module.

**Table 6:** PPG signal properties and mean HR parameters for two measurement conditions inside the MRI device for groups of male and female tested persons separately.

Parameter/Condition ^(A)^	*PPG-EP* (CD&NS/CD&RS)		*PPG-PS1* (CD&NS/CD&RS)		*PPG-BLE* (CD&NS/CD&RS)	
	Male	Female	Male	Female	Male	Female
*PPG*_RANGE_ [%]	55/52	49/47	50/48	45/42	45/43	41/40
*HP*_RIPP_ [%]	13/14	15/16	15/17	18/20	15/16	17/18
*HR*_VAR_ [%]	1.8/1.9	2.4/3.2	2.9/4.7	3.4/6.2	2.7/3.8	3.1/5.5

^(A)^ Mean values both hands together.

## Data Availability

The data supporting reported results are not readily available because they can be used only for research purposes and our paper must be cited where our data are used. Requests to access the data should be directed to the corresponding author Jiří Přibil (umerprib@savba.sk).
